# Progressive quadriparesis in a young woman due to a spinal perimedullary arteriovenous fistula (PMAVF type IVa) successfully treated with endovascular therapy: A case report

**DOI:** 10.1016/j.radcr.2025.05.027

**Published:** 2025-06-03

**Authors:** Fitri Damayanti, Ashari Bahar, Wijoyo Halim

**Affiliations:** a Department of Neurology, Faculty of Medicine, Division of Neurointervention and Endovascular Therapy, Hasanuddin University, Hasanuddin University Hospital/Brain Centre, Dr. Wahidin Sudirohusodo General Hospital, Makassar, South Sulawesi, Indonesia; b Department of Neurology, Faculty of Medicine, Alkhairaat University, Palu, Indonesia

**Keywords:** Peri-medullary arteriovenous fistula, Transarterial embolization, Myelopathy, Neurointervention, Case report

## Abstract

A perimedullary arteriovenous fistula (PMAVF) is a rare spinal vascular malformation that can lead to progressive neurologic deficits. We report a case of a 23-year-old woman presenting with progressive quadriparesis over 6 months. Initial spinal MRI revealed serpiginous flow voids from the cervical to the lumbar region, indicating abnormal vessels. Subsequent spinal angiography confirmed a PMAVF type IVa arising from the anterior radiculomedullary artery at the tenth thoracal (T10) level, shunting directly into a dilated perimedullary venous system. Transarterial embolization using ethylene-vinyl alcohol copolymer (Onyx) successfully occluded the fistula without compromising normal arterial flow. The patient showed marked neurologic recovery, achieving near-complete motor improvement by the seventh day postprocedure. PMAVF is classified as a type IV spinal vascular malformation and may cause severe neurologic symptoms due to venous hypertension, congestion, or hemorrhage. Early diagnosis is crucial, as endovascular embolization offers a minimally invasive treatment option with a favorable safety profile. This case illustrates the importance of timely recognition and intervention in PMAVF, underscoring the potential for significant functional improvement following endovascular therapy.

## Introduction

Spinal vascular shunts, including arteriovenous malformations (AVMs) and arteriovenous fistulas (AVFs) are rare and account for only 3%-4% of spinal lesions, posing challenges in imaging and therapeutic management [1]. The latest AFVs classification system was developed by Takai (2016), which divides into 5 categories. One of the very rare cases is perimedullary arteriovenous fistula (PMAVF) [2]. The etiology of spinal AVFs is multifactorial and remains incompletely understood. Some hypotheses suggest a congenital disposition where a latent vascular anomaly is triggered later in life [3]. Others suggest that acquired mechanisms involve chronic inflammation, microtrauma, or degenerative changes [2,4]. They disrupt normal spinal cord circulation by creating abnormal arteriovenous connections, which can progressively compromise neurological function [3,5]. Although their course is characteristically insidious, early signs such as limb weakness, intermittent cramping, or transient numbness may be easily overlooked [6,7]. Consequently, many patients do not receive a timely diagnosis, allowing the underlying pathology to advance unchecked. In recent decades, treatment methods with endovascular techniques have been developed that provide good outcomes in the treatment of PMAVF [8]. We presented a young female patient with PMAVF type IVa who was successfully treated with endovascular interventional methods.

## Case presentation

A 23-year-old woman was referred to the neurology department with progressive weakness affecting all 4 limbs. Initially, she experienced gradual onset of weakness and cramps localized to the right upper and lower extremities, which had been progressing over the past 6 months. This was associated with neck pain radiating to the lower back and right groin. The pain was exacerbated by physical activity and relieved by rest. One month prior to presentation, the weakness had worsened and began to involve the left side as well, with greater weakness noted in the left leg compared to the left arm.

There was no history of trauma, fever, speech disturbances, headache, altered mental status, or systemic illnesses such as diabetes mellitus or hypertension. The patient also denied any history of chronic cough, prior tuberculosis treatment, or urinary and bowel complaints. There was no known family history of vascular or neurological disorders.

Vital signs were within normal limits. Neurological examination revealed asymmetrical quadriparesis with Medical Research Council (MRC) grade 4 strength in the right leg, and grade 5- in the right arm, left leg, and left arm. Increased muscle tone was noted in all 4 limbs. Pathological reflexes including bilateral Hoffmann’s, Babinski’s, and Chaddock’s signs were present. Deep tendon reflexes, particularly the Achilles reflexes, were brisk bilaterally, and ankle clonus was elicited on both sides. Sensory examination demonstrated hypesthesia in a stocking-glove distribution extending up to the C2 dermatome.

**Fig. 1:**
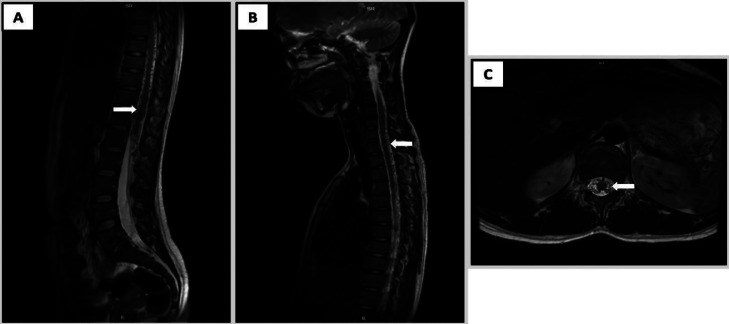
Pretreatment magnetic resonance (MR) images. (A) Sagittal T2-weighted MR image of the thoraco-lumbar spine showing vermiform vascular shadows extending from the thoracic to lumbar segments and spinal cord edema; (B) Sagittal T2-weighted MR image of the cervico-thoracal segment showing perimedullary flow void (C) Axial T2-weighted MR image showing dilatation of perimedullary vein in thoracal level.

**Fig. 2:**
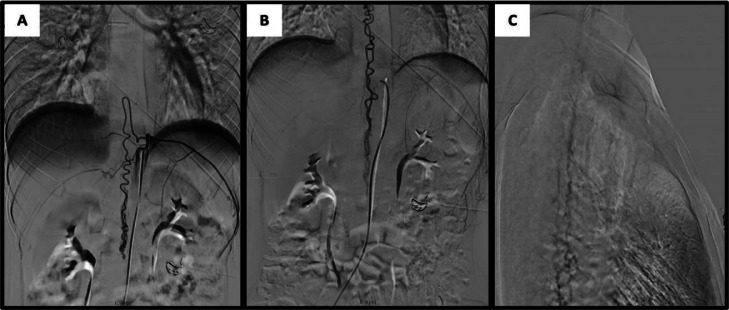
Digital subtraction angiography of the spine. (A) An anteroposterior (AP) angiogram of the left T10 segment shows a fistula fed from left great anterior radicullomedullary artery of Adamkiewicz to the anterior spinal artery (ASA). (B) Venous phase showing dilated perimedullary venous drainage in thoraco-lumbar level (AP view). (C) Venous phase in cervico-thoracal level (lateral view).

Routine laboratory investigations were unremarkable, showing no evidence of infection, inflammation, or metabolic derangement. Chest radiography was normal. However, spinal magnetic resonance imaging (MRI) revealed serpiginous flow voids extending from the C2 to L2 vertebral levels, consistent with a tangle of abnormal perimedullary vessels suggestive of a spinal arteriovenous fistula (Fig. 1). Under local anesthesia, vascular access was obtained via puncture of the right common femoral artery, followed by diagnostic spinal angiography. Angiogram showed a fistula connection between the anterior radiculomedullary artery, originating from the left tenth thoracic segmental artery (T10) and the anterior spinal artery (ASA), with drainage into the dilated perimedullary vein, confirming a type IVa perimedullary arteriovenous fistula (PMAVF) (Fig. 2).

**Fig. 3:**
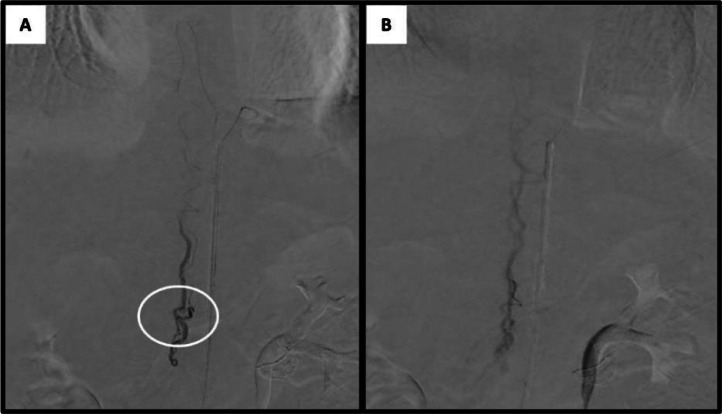
Selective spinal angiography using microcatheter. (A) The fistula during arterial phase. (B) The draining vein.

**Fig. 4:**
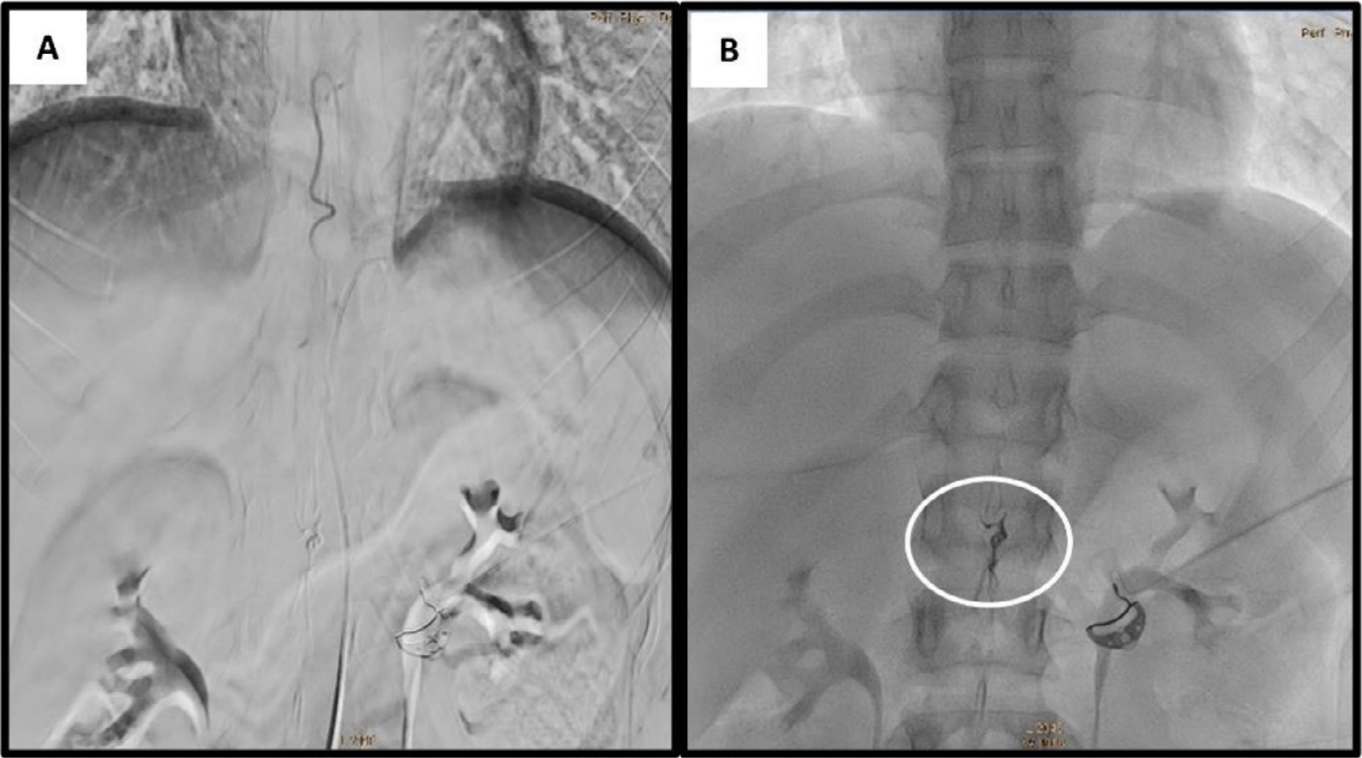
Post embolization angiography. (A) The arteriovenous fistula was completely occluded after embolization using Onyx18. (B) Dense Onyx18 cast ensures complete fistula occlusion.

Subsequent super-selective spinal angiography under general anesthesia was performed using a microcatheter, providing clearer delineation of the fistula (Fig. 3). We evaluated the case and determined that embolization with ethylene vinyl alcohol copolymer (EVOH) (Onyx18, Medtronic Inc.) was the most appropriate treatment. A Marathon microcatheter (Medtronic Inc.) was advanced over a Mirage microwire (Medtronic Inc.) to a position just proximal to the fistula. Onyx18 and DMSO were prepared in 1 mL syringes, and the dead space of the microcatheter was filled with DMSO before Onyx18 was slowly injected by hand over 90 seconds to displace the DMSO. Under continuous fluoroscopic guidance, Onyx18 was injected into the fistula using the "thumb-tapping" technique. When the embolic agent progressed antegrade, the injection can be continued until complete obliteration of the lesion is achieved. Some reflux occurred, leading to the formation of a “plug,” which subsequently facilitated antegrade flow of the embolic agent. Complete obliteration of the fistula was confirmed angiographically, and the radiopaque cast of Onyx18 was visualized (Fig. 4).

**Fig. 5:**
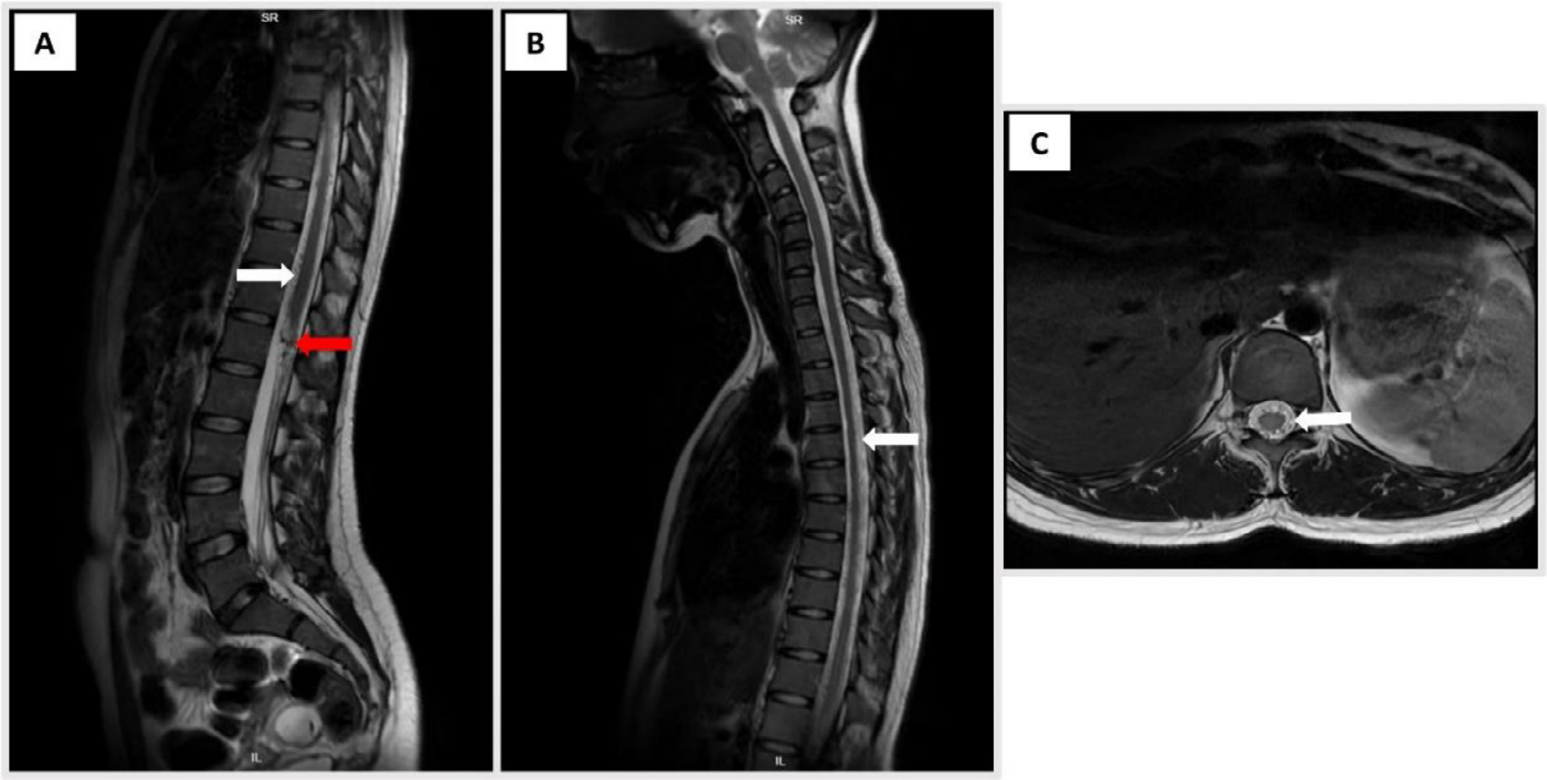
Postembolization MRI. (A) Sagittal T2-weighted MR image of the thoraco-lumbar spine showing a decrease in spinal cord edema, minimal dilated vascular flow voids around the spinal cord (white arrow), and Onyx cast (red arrow). (B) Sagittal T2-weighted MR image of the cervico-thoracic segment showing decreased perimedullary flow void compared to before. (C) Axial T2-weighted MR image showing minimal dilatation of perimedullary vein in thoracal level.

Postprocedural follow-up on day 3 showed notable improvement in motor strength. By day 7, the patient was able to ambulate independently, although mild residual weakness persisted in the right leg. She was subsequently referred for medical rehabilitation. One month postembolization, follow-up MRI demonstrated marked reduction in perimedullary vein dilatation and resolution of spinal cord edema (Fig. 5).

## Discussion

A perimedullary arteriovenous fistula (PMAVF) is a vascular malformation located on the surface of the spinal cord, usually an anterior or lateral surface and is characterized by a direct arteriovenous shunt without an intervening nidus. The incidence of PMAVF is approximately 8%-19% of all spinal malformations. While thoracic and lumbar levels predominate in many reports, no spinal region is exempt. It is often not detected in time, as they do not initially present with striking symptoms [3,9,10].

The classification of AVF was first revealed by Di Chiro in 1971, who divided it into 3 types of AVF: single coiled vessel type I, glomus type II, and juvenile type III. Over time, this classification was refined by Takai in 2016 and divided AVF into 5 types: dural AVF (type I), intradural intramedullary glomus AVM (type II), intradural intramedullary juvenile AVM (type III), perimedullary AVF (type IV) and extradural AVF (type V). Perimedullary AVF is further subdivided into subtypes IVa, IVb and IVc, based on the number of feeders, the pattern and size of venous drainage, and the presence or absence of collateral posterior spinal artery (PSA) supply [2,11]. In this case, the single feeder artery originated from the radiculomedullary artery of the T10 segment and then passed through the ASA before finally forming a fistula, so we classified it as type IVa.

PMAVF type IVa is more common in the elderly (approximately 35 years old), compared to type IVb and type IVc which are more common in children. It is generally caused by idiopathic causes and some cases are stated to be caused by trauma or develop after spinal surgery [1,3,8,12]. The incidence of PMAVF type IVa is very rare among all cases of spinal vascular shunts compared to other types. According to the literature, type IVa PMAVF is more common in older age, but our patient was only 23 years old when diagnosed. This brings to light the fact that type Iva PMAVF can also be experienced by younger people.

The main pathophysiology of PMAVF is the direct conduit between arteries and veins without passing through the regulatory capillary network [1,2]. In the absence of capillary resistance, high-pressure arterial flow enters the venous system, resulting in venous hypertension [9]. This dysregulated circulation impairs perfusion, restricts oxygen delivery, and initiates an ischemic cascade. Over time, demyelination and axonal degeneration will occur, leading to progressive neurological deficits [13].

Nonspecific clinical symptoms often result in delayed diagnosis and treatment. Common complaints include back pain, fatigue in the lower limbs, or mild sensory disturbances often attributed to more prevalent spinal disorders like herniated discs or spondylosis [12,14]. Slowly progressive asymmetric myoradiculopathy of the conus and cauda equina was the most common symptom in most patients (91%). As the pathology progresses, symptoms such as hyperreflexia and sphincter dysfunction may also appear [3,11]. In our patient, motor weakness was felt gradually since 6 months and initially only in the right leg. However, the condition gets worse and is eventually followed by weakness in the other extremities. There were sensory disturbances up to the C2 dermatome area, but fortunately, there were no complaints of urination or defecation.

MRI typically provides the initial clues, revealing serpiginous flow voids, cord edema, and abnormal pial vessels that raise suspicion for a fistula. However, spinal angiography remains the definitive diagnostic modality, precisely delineating arterial feeders, shunting pathways, and venous drainage anomalies [5,13,15]. Type IVa PMAVF is the least common type IV, typically formed by a small and single-vessel fistula supplied by a normal caliber anterior spinal artery (ASA) or radiculomedullary arteries and mildly dilated perimedullary vein along the surfaces of the conus medullaris or filum terminale [1,11,16]. The spinal MRI in our patient showed a serpiginous flow void extending from C2 to L2 and a tangle of abnormal blood vessels flowing through the spinal cord, which was suspected to be a spinal AVF. Then, selective angiography confirmed a fistula supplied from the anterior radiculomedullary artery at the T10 level and then through the anterior spinal artery, with a network of dilated veins extending up to the C5 level. One month after embolization of the fistula, a control spinal MRI was performed, and there was significant improvement in the MRI image. Spinal cord edema was minimal, and there was much less dilatation of the perimedullary veins.

PMAVF is more difficult to treat than spinal dural AVF (SDAVF) due to its complicated anatomy and physiology. Type IVa PMAVF is generally treated with microsurgery, because of difficulty of navigating into fistulas with small diameter. However, advent of endovascular therapy has transformed treatment strategies. Open surgery, once the standard, has now been replaced by precise, minimally invasive and clinically effective embolization. In elderly patients, obese patients, or patients with comorbidities who have a higher risk of postoperative complications such as infection and deep vein thrombosis, endovascular therapy is a suitable option [8,12,17].

Endovascular treatment with ASA occlusion is considered to have a high risk of ischemic complications, which can lead to paralysis when spinal angiography shows that the feeder is a branch of the ASA or Adamkiewicz artery. Several previous studies have reported that in ventral lesions, it is difficult to perform surgical resection with anterior and posterior approaches due to the great risk of spinal cord injury. The challenge to performing type IVa PMAVF embolization is the small diameter of the feeding artery which makes it difficult to optimally position the catheter tip for super selective fistula embolization. Rapid advances in endovascular techniques using microcatheters that are very soft and smaller than the ASA have made it possible to embolize only the lesion without damaging the ASA [8,12].

In this case, endovascular embolization using a liquid embolic agent (Onyx) was performed, targeting the anomalous arterial feeder. Inject Onyx into the feeder artery as close as possible to the fistula until the proximal part of the draining vein and the fistula are occluded. Onyx injection is easier to control, and the procedure does not require rapid catheter withdrawal immediately after embolization, making Onyx considered more advantageous than N-butyl 2 cyanoacrylate (NBCA). Onyx is equally effective in treating peripheral lesions, whether used alone or in combination with other embolic agents. Its advantages include unique physical properties such as high viscosity, nonadherence to surfaces, delayed polymerization, adaptability to various blood vessel shapes, and excellent fluoroscopic visibility. However, after injection of Onyx, it takes some time for the proximal plug to form, so there is a risk of longer fluoroscopy time and radiation exposure [8,13].

Several factors that can determine patient outcome include the duration of clinical symptoms, the severity of weakness or other symptoms, and the success of the initial procedure to close the fistula. Fast and accurate diagnosis is also a determining factor in the success of treatment. The time between the onset of initial symptoms and the speedy access to endovascular therapy or surgery will provide better outcomes. A better prognosis was also obtained in patients with less severe symptoms prior to intervention and subsequent rehabilitation [5,18]. Clinical outcomes of PMAVF were relatively better than intramedullary arteriovenous fistula after either surgical or endovascular treatment. The complete obliteration rate was approximately 74%-88%, the clinical improvement rate was approximately 68% in adults and 80%-82% in the pediatric group, and the complication rate was 0%-11% [16,17]. Early treatment often correlates with significant neurological recovery, as observed in this case, where motor function improved substantially within days.

This case was rarely discussed in previous references due to its rarity, so it is interesting to discuss and may enrich the review of references on type IVa PMAVF. That this case may contribute valuable clinical and imaging data to the limited literature on type IVa PMAVF, especially in young adults. This case also underscores the important role of early diagnosis, advanced imaging, and evolving endovascular techniques in optimizing outcomes for complex spinal vascular lesions.

## Conclusion

Perimedullary arteriovenous fistula (PMAVF) represents a critical yet potentially treatable cause of progressive myelopathy, highlighting the importance of prompt diagnosis and timely endovascular intervention. MRI, in conjunction with spinal angiography, plays a central role in defining vascular anatomy and guiding targeted therapy. Endovascular embolization has emerged as a primary treatment with high efficacy, but its success strongly correlates with the speed of intervention. Therefore, a multidisciplinary approach proves essential—neurologists, radiologists, interventional specialists, and physical medicine and rehabilitation specialists collaborate to address these complex vascular anomalies.

## Ethical statement

The work has been performed by the ethical standards laid down in the 1964 Declaration of Helsinki and its later amendments. This case report was conducted by ethical standards, and patient consent was obtained for publication.

## Author contributions

All authors contributed to the writing of the manuscript and approved the final version of the manuscript for publication in the journal.

## Declaration of generative AI and AI-assisted technologies in the writing process

Use of artificial intelligence (AI)-assisted technology for manuscript preparation: The authors confirm that there was no use of artificial intelligence (AI)-assisted technology for assisting in the writing or editing of the manuscript and no images were manipulated using AI.

## Patient consent

I, the undersigned, hereby give my consent for the case report described above, including any images and clinical details, to be published in a medical journal. I understand that:

• My name and initials will not be published, and every effort will be made to ensure my anonymity.
• The information may be published in print and online and may be viewed by readers worldwide.
• The information may also be used in presentations, medical education, or research.

I have been informed that I will not receive any compensation for the use of this information.

I have read and understood the above information and give my consent freely.
